# Association of Single Nucleotide Polymorphism *LEP-R* c.668A>G (p.Gln223Arg, rs1137101) of leptin receptor gene with endometrial cancer

**DOI:** 10.1186/s12885-021-08620-y

**Published:** 2021-08-16

**Authors:** Jan Bieńkiewicz, Hanna Romanowicz, Miłosz Wilczyński, Grzegorz Jabłoński, Anna Stepowicz, Anna Obłękowska, Andrzej Malinowski, Beata Smolarz

**Affiliations:** 1 Department of Operative Gynecology, Endoscopy and Gynecologic Oncology, Polish Mother’s Memorial Hospital-Research Institute, Lodz, Poland; 2 Department of Clinical Pathology, Polish Mother’s Memorial Hospital – Research Institute, Lodz, Poland; 3 Department of Obstetrics, Perinatology and Gynecology, Polish Mother’s Memorial Hospital-Research Institute, Lodz, Poland; 4 Department of Operative and Endoscopic Gynecology, Medical University of Lodz, Lodz, Poland; 5 Laboratory of Cancer Genetics, Department of Clinical Pathology, Polish Mother’s Memorial Hospital-Research Institute, Lodz, Poland

**Keywords:** Single nucleotide polymorphism, p.Gln223Arg, *LEP-R*, c.668A>G, rs1137101, Endometrial cancer, Uterine leiomyomas, Uterine fibroids, Leptin, Obesity, Adipokines

## Abstract

**Background:**

The aim of this study was to analyze the frequencies of genotypes and alleles of Single Nucleotide Polymorphism (SNP) *LEP-R* c.668A>G **(**p.Gln223Arg, rs1137101) of leptin receptor gene and to assess the influence this DNA marker has on endometrial cancer (EC) with respect to total body fat content.

**Methods:**

The study comprised 120 patients treated for endometrial cancer and 90 controls treated for uterine fibroids. In total, 210 patients were included in this research. DNA was isolated from archival post-operative specimens. Polymerase Chain Reaction – Restriction Fragment Length Polymorphism was employed to analyze the SNP.

**Results:**

In this paper we have demonstrated that heterozygous genotype AG of SNP *LEP-R* c.668A>G **(**p.Gln223Arg, rs1137101) is statistically less frequent in women with endometrial cancer (EC) than in controls: 33 *versus* 57%, respectively. Similarly, this heterozygous genotype is statistically significantly less frequent in obese (BMI > 30) women with EC than in lean controls (BMI < 25): 30 *versus* 63%, respectively.

**Conclusions:**

AG polymorphic variant of SNP *LEP-R* c.668A>G (p.Gln223Arg, rs1137101) in *LEP-R* may be considered a protective factor in the development of endometrial cancer.

## Background

Obesity and endometrial cancer (EC) are two heavily associated morbidities in developed countries. Should one employ BMI as a sole measure of total body fat content, obese women (30<BMI<35) have a 2,6-fold elevated risk of developing EC, whereas in severely obese individuals (BMI>35) this hazard boosts up to 4,7-fold compared to ones with weight within normal range (BMI<25) [1].

Obesity mostly develops as a consequence of chronic imbalance between energy intake and its expenditure. Leptin, the ‘satiety hormone’, is the one of a major value in regulating food ingestion as it inhibits the sensation of hunger. Among various until-now analyzed adipokines, the role of leptin in the development of EC has been well established: according to reliable data, high levels of leptin are directly correlated with the risk of this malignancy [2]. As the synthesis of all proteins in human body strictly depends on the transcription and mRNA, leptin – as an example of a highly polymorphic protein – is a particularly susceptible product of polymorphic genome variants: PubMed database displays more than 220 results using the search criterion: [leptin + polymorphism] (as of March 2021).

In our previous paper we have analyzed the role of single nucleotide polymorphism (SNP) -2548A/G of *LEP* in EC. Our results revealed that genotype AG of SNP − 2548A/G may reduce the risk of developing EC, whereas allele A, independently, could be a risk factor of this malignancy [3]. For all leptin’s metabolic activity is mediated through specific receptors, their polymorphisms have also been studied. There are numerous SNPs found in *LEP-R,* among which rs1137101 A>G polymorphism (p.Gln223Arg) seems to be the one most thoroughly studied in terms of its association with neoplastic transformation [4]. According to three large meta-analyses, the role of rs1137101 A>G polymorphism (p.Gln223Arg) in overall cancer susceptibility is rather denied [4–6]. However – to our best knowledge – the correlation between this SNP and endometrial cancer has not yet been investigated.

Encouraged by the results of our earlier study on − 2548A/G and taking into consideration the role the abovementioned SNP plays in cancer risk [6–8], we decided to go further and investigate and elucidate, if polymorphic variants of *LEP-R* may also alter the hazard of endometrial cancer. In this study we have aimed to determine the influence of SNP rs1137101 A>G polymorphism (p.Gln223Arg) of *LEP-R* on its allele-specific expression in women with endometrial cancer with further analysis of its interrelation with obesity.

## Methods

### Patients

In this research we have used a similar design as in our previous paper on SNP -2548A/G of *LEP* in endometrial cancer [3]. The test group (TG) included 120 women treated surgically for the before mentioned disease in the Department of Operative Gynecology, Endoscopy and Gynecologic Oncology, Polish Mother’s Memorial Hospital-Research Institute, Lodz, Poland in the period: 2003–2012. Within this group an additional division was introduced in order to stratify the patients into: lean, overweight and obese, according to Body Mass Index (BMI), which was used here as a marker of total body fat content. In consequence, three subgroups within TG were formed:

Test Group 1 (TG1) – BMI<25 (*n*=40).

Test Group 2 (TG2) – 25≤BMI < 30 (*n*=40).

Test Group 3 (TG3) – BMI≥30 (*n*=40).

90 age-matched females treated surgically in the Department for uterine leiomyomas were selected as Controls (C). Alike, these individuals were stratified according to BMI to obtain three corresponding subgroups:

Controls 1 (C1) – BMI<25 (*n*=30).

Controls 2 (C2) – BMI 25≤BMI<30 (*n*=30).

Controls 3 (C3) – BMI≥30 (*n*=30).

DNA of both Test Group and Controls was isolated from archival postoperative specimens stored in paraffin blocks in the Department of Clinical Pathology, Polish Mother’s Memorial Hospital – Research Institute, Lodz, Poland. Since diabetes mellitus and glucose intolerance (GI) – as pathologies impacting the metabolism of carbohydrates – are believed to correlate with adipokines and their expression [9–11], patients suffering from any of the abovementioned conditions were excluded from the study in order to avoid potential bias. All tissue and genetic assays were performed in the Laboratory of Cancer Genetics, Department of Clinical Pathology, Polish Mother’s Memorial Hospital – Research Institute, Lodz, Poland.

### DNA isolation

Tissue specimens were originally fixed in formaldehyde and then embedded in paraffin and under such conditions they were stored in the archives of Department of Clinical Pathology. To obtain the DNA for research, the paraffin blocks were microtome-sectioned at the thicknesses of 5 μm, transferred to Eppendorf® micro test tubes and shaken five times with xylene with a 3-min-long centrifugation (14,000 RPM) after each shaking. The sediment was lavaged in 96% ethanol and again centrifuged for 3 min and dried in 37 °C. DNA was extracted from the material by DNeasy Blood & Tissue Kit (Qiagen, Germany) according to the manufacturer’s instruction. DNA specimens were then stored for research in − 20 °C.

### Genotype determination

Polymerase Chain Reaction – Restriction Fragment Length Polymorphism was employed to analyze the Single Nucleotide Polymorphism (SNP) *LEP-R* c.668A>G (p.Gln223Arg, rs1137101) of leptin receptor gene in the specimens. The reaction was performed in a final volume of 50 μl of reaction mixture which contained: 100 ng of genomic DNA, 5 μl PCR buffer (TaKaRa, Japan), 4 μl dNTP (10 mM, TaKaRa, Japan), 1 unit of Taq Polymerase (TaKaRa, Japan) and 0,5 μl of each primer (10 mM, Polgen, Lodz, Poland). Deionized H_2_O was added. The amplification was completed in Thermal Cycler PTC-100 TM (MJ Research, INC, Waltham, MA, USA) in conditions as follows: initial denaturation in 94 °C (3 min) which was followed by 35 cycles of: denaturation in 94 °C (60 s), hybridization with starters in 65 °C (60 s) and finally augmented to 72 °C (90 s). Synthesis was concluded in 72 °C (7 min). Following starters were used:

forward: 5′-AAA CTC AAC GAC ACT CTC CTT-3′.

reverse: 5′-TGA ACT GAC ATT AGA GGT GAC-3′.

The PCF-RFLP product (20 μl of reaction mixture) was incubated overnight with 1 unit of restriction enzyme MspI (Fermantas) in 37 °C.

### Electrophoresis

The reaction products were electrophoresed in a 2% agarose gel (AppliChem GmbH, Darmstadt, Germany). BIORON DNA Ladder 50 bp (Bioron GmbH, Ludwigshafen, Germany) was used as mass ruler. Electrophoresis was performed in BIOMETRA unit applying 6 V/cm electric field. Visualization followed after ethidium bromide staining (10 mg/ml, AppliChem GmbH, Darmstadt, Germany). Gel was analyzed under UV conditions using microDOC unit (Major Science, Syngen). Fragments of following lengths were obtained:

1. 216 bp, 134 bp and 82 bp – representing heterozygous genotype AG
2. 216 bp – representing homozygous genotype AA
3. 134 bp and 82 bp –representing homozygous genotype GG

### Statistical analysis

First comparison was made between the two main groups [Test Group (*n*=120) and Controls (*n*=90)] without any respect to total body fat content (*i.e.* BMI). Consequently, all 6 subgroups (lean, overweight and obese) were compared. The genotype and allele distribution, as well as the establishment of the compatibility with Hardy-Weinberg rule, were assessed using χ^2^ test. Similarly, differences between distributions in subgroups were evaluated by χ^2^ test. Both genotypes’ and alleles’ impact on the risk of disease was assessed by odds ratio (OR) with 95% confidence interval, adjusted to the logistic regression model. Wild type of genotype and allele were used for reference. Statistical significance was confirmed at *p*<0,05. STATISTICA 11 (StatSoft, Poznań, Poland) software was used for statistical analysis of data.

## Results

**Fig. 1:**
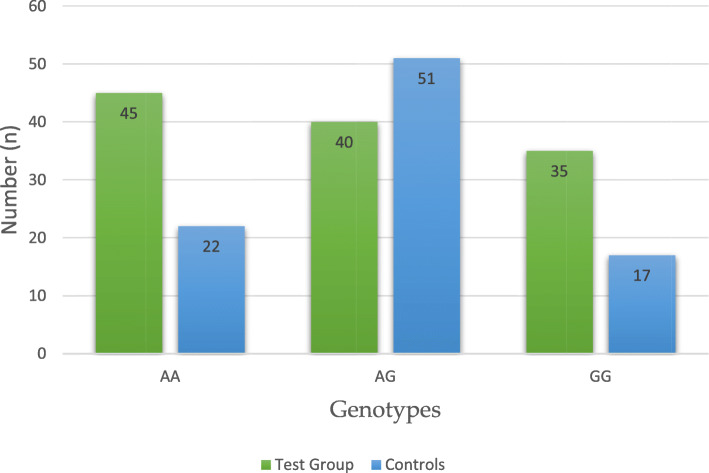
Distribution of genotypes of SNP *LEP-R* c.668A>G (p.Gln223Arg, rs1137101) in Cases and Controls

**Fig. 2:**
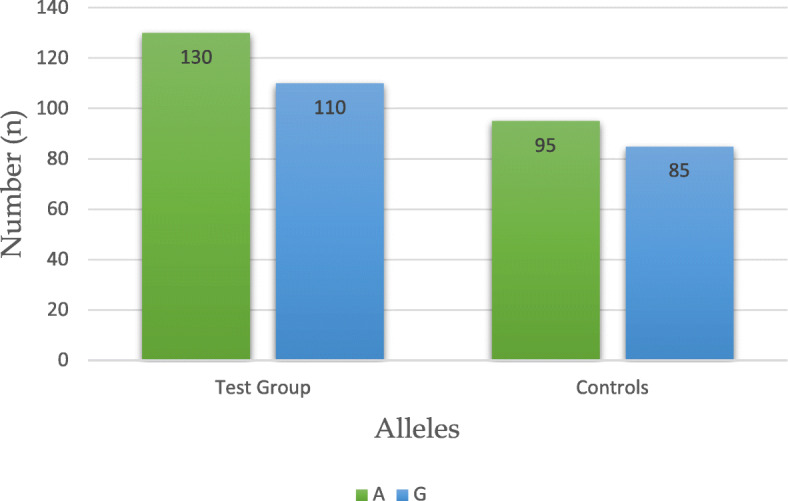
Distribution of alleles of SNP *LEP-R* c.668A>G (p.Gln223Arg, rs1137101) in Cases and Controls

**Fig. 3:**
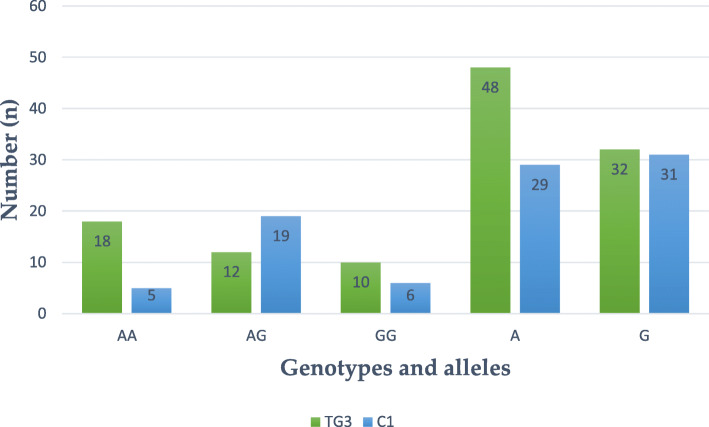
Distribution of genotypes and alleles of SNP *LEP-R* c.668A>G (p.Gln223Arg, rs1137101) in obese Cases (TG3) and lean Controls (C1)

Table 1 presents the total distribution of genotypes and alleles of Single Nucleotide Polymorphism (SNP) *LEP-R* c.668A>G (p.Gln223Arg, rs1137101) in both Test Group (*n*=120) and Controls (*n*=90), without respect to the total body fat content (*i.e. *BMI). We have found that heterozygous genotype AG of this DNA marker is statistically significantly less frequent in women with endometrial cancer (EC) than in Controls: 33 *versus* 57%, respectively. The distribution of genotypes and alleles of the studied SNP is graphically illustrated in Figs. 1 and 2. Subsequently, intercorrelations between the BMI-stratified subgroups were analyzed. An interesting finding was revealed upon juxtaposition of obese endometrial cancer patients (TG3) with lean controls (C3), where heterozygous genotype AG was found to be statistically less frequent in the former group than the latter (30 *versus* 63%, respectively, OR, 0,18, *p* 0,009). Table 2 presents the abovementioned distribution and finding. Graphic illustration is provided in Fig. 3. No statistically significant results were observed in comparisons between other BMI-stratified subgroups.

**Table 1:** Distribution of genotypes and alleles of SNP *LEP-R* c.668A>G (p.Gln223Arg, rs1137101) in Test Group and Controls (in total)

	1		2	
	Test Group (*n*=120)		Controls (*n*=90)	
	*n*	(%)	*n*	(%)
AA	45	38	22	24
AG	40	33	51	57
GG	35	29	17	19
A	130	54	95	53
G	110	46	85	47
OR (95% PU)^a^				
	1–2	*p*^b^		
AA	1.00 Ref.^c^			
AG	**0.38 (0.20–0.74)**	**0.006**		
GG	1.01 (0.47–2.18)	0.862		
A	1.00 Ref.			
G	0.95 (0.64–1.40)	0.862		

^a^ Odds ratio analysis [OR – odds ratio, CI – confidence interval 95%]

^b^ For the departure from Hardy-Weinberg equilibrium

^c^ Reference: wild allele

**Table 2:** Distribution of genotypes and alleles of SNP *LEP-R* c.668A>G (p.Gln223Arg, rs1137101) in TG3 and C1

	1		2	
	TG3 (=40)		C1 (*n*=30)	
	*n*	(%)	*n*	(%)
AA	18	45	5	17
AG	12	30	19	63
GG	10	25	6	20
A	48	60	29	48
G	32	40	31	52
OR (95% CI)^a^				
	1–2	*p*^b^		
AA	1.00 Ref.^c^			
AG	**0.18 (0.05–0.59)**	**0.009**		
GG	0.46 (0.11–1.90)	0.236		
A	1.00 Ref.			
G	0.62 [0.31–1.22]	0.230		

^a^ Odds ratio analysis [OR – odds ratio, CI – confidence interval 95%]

^b^ For the departure from Hardy-Weinberg equilibrium

^c^ Reference: wild allele

## Discussion

This study revealed statistically significant differences in the distribution of genotypes of SNP *LEP-R* c.668A>G (p.Gln223Arg, rs1137101) between Test Group and Controls. We found that the heterozygous polymorphic genotype of the studied DNA marker is statistically significantly less frequent in women with endometrial cancer which may suggest its protective significance in this malignancy. Interestingly, no such correlation could be observed in homozygous polymorphic variant. This point can be explained and justified by the limitations of our study which will be discussed further. Moreover, we have revealed, that there is a correlation in the distribution of genotypes of this DNA marker in obese patients with endometrial cancer (TG3) and in lean controls (C1). In both comparisons the heterozygous polymorphic variant AG was significantly less frequent in EC patients, which allows to draw a conclusion that heterozygote AG of the SNP may be a protective factor concerning EC. However, one has to take into consideration the obvious limitations of our study. Firstly, the exact interrelation between SNP *LEP-R* c.668A>G (p.Gln223Arg, rs1137101) and uterine leiomyomas, to our best knowledge, has not yet been established. One can expect there is no such correlation, as uterine fibroids’ pathogenesis (contrary to diabetes mellitus and glucose intolerance) reaches to quite different origins than metabolism of carbohydrates [12] and leptin should not play any role here. Until now, solely serum leptin levels in patients with uterine leiomyomas have been studied, but the data is inconsistent [13, 14]. Still, our previous research [3] provides the only available data on leptin gene polymorphism and uterine fibroids with a conclusion that there is no such correlation. Regrettably, in current research, contrary to our original intention, we could not examine disease-free controls as the contract between our institution and BioBank (which had provided samples of disease-free individuals previously) has expired. Secondly, the size of our groups may be quantitively unsatisfactory to draw definite conclusions regarding genetic phenomena in cancer [15, 16]. Finally, obesity as such is strongly interconnected with adipokines and, likely, the genetic phenomena that interact with them [17–20]. As the second statistically significant finding in this research was observed upon confronting obese patients with endometrial cancer (TG3) with lean controls (C1) one can easily challenge the overall study conclusion and question, if the results have not been biased by obesity itself as an obvious comorbidity of TG3 cancer patients.

## Conclusions

In our study we have demonstrated that Single Nucleotide Polymorphism (SNP) *LEP-R* c.668A>G (p.Gln223Arg, rs1137101) could play a role in the risk of endometrial cancer. We conclude, that the polymorphic heterozygous variant AG of the SNP may be considered a protective factor in the development of endometrial cancer, also in obese individuals. Nonetheless, taking into account clear limitations of our research, further studies on larger groups are warranted to derive more definite conclusions on the impact this genetic marker has on endometrial cancer.

## Data Availability

All data available at Institution.

## Abbreviations

BMI: Body mass index
C: Controls
EC: Endometrial cancer
GI: Glucose intolerance
TG: Test group
SNP: Single nucleotide polymorphism
